# Downregulation of SEPTIN5 inhibits prostate cancer progression by increasing CD8^+^ T cell infiltration

**DOI:** 10.7150/ijbs.76573

**Published:** 2022-10-17

**Authors:** Ming Chang, Yu He, Cong Liu, Risheng Lin, Xin Huang, Dongcuan Liang, Jian Zhang, Yi Lu

**Affiliations:** 1 School of Medicine, Southern University of Science and Technology, Shenzhen, Guangdong 518055, China.; 2 Guangdong Provincial Key Laboratory of Cell Microenvironment and Disease Research, Shenzhen, Guangdong 518055, China.; 3 Aurora Discovery Inc, Foshan, Guangdong 528303, China.

**Keywords:** prostate cancer, SEPTIN5, immune infiltrating, CD8^+^ T cells, chemokine, cytokine.

## Abstract

**Background:** Prostate cancer (PCa) is one of the most common carcinomas in men, and aberrant expression of SEPTIN5 (SEPT5) has been detected in PCa tissues. However, the role of SEPT5 in PCa is still unclear. In this study, we attempted to explore the expression changes, clinical relevance and immunomodulatory function of SEPT5 in PCa.

**Methods:** The expression and clinical significance of SEPT5 were evaluated based on bioinformatic analysis and were verified by western blotting, real time PCR and IHC. Allograft mouse models were used to assess the role of SEPT5 on PCa tumour formation and immunomodulatory function. Mass cytometry and IHC were used to determine the effects of SEPT5 on immune cell infiltration, especially CD8^+^ T cell infiltration. Correlations between SEPT5 expression and cytokine gene expression were analyzed based on TCGA and DKFZ datasets. RNA-seq and chemokine array were performed to confirm the effects of SEPT5 on cytokine production.

**Results:** High SEPT5 expression was found in PCa and was associated with PCa prognosis. Importantly, downregulation of SEPT5 inhibited PCa progression* in vivo*. In addition, SEPT5 expression was negatively correlated with immune infiltrating cell levels, chemokine and cytokine gene expression associated with CD8^+^ T cell infiltration and activation. Downregulation of SEPT5 increased the proportion of immune cells, especially CD8^+^ T cells, in tumour tissue. Both the expression of CCL5, CXCL5, CXCL9, CXCL10 and INFGR1 were elevated in mRNA and protein levels after SEPT5 knockdown.

**Conclusions:** In summary, downregulation of SEPT5 inhibited PCa progression, which may be mediated by increasing immune cell infiltration levels, especially CD8^+^ T cells, by promoting the production of IFNG-inducible chemokines and cytokines expression associated with immune cell infiltration. Our findings suggest that SEPT5 may serve as a prognostic biomarker of PCa and may be a target molecule to enhance the efficacy of immunotherapy for PCa in the future.

## Introduction

Prostate cancer (PCa) was one of the most common cancers in men in 2020 and was the most frequently diagnosed cancer in 112 countries, especially in the USA and Europe 1. In 2022, it is estimated that PCa will account for 27% of diagnosed cancer cases and 11% of deaths in men in the USA 2. However, the pathogenic mechanism of PCa is still unclear, which undoubtedly increases the difficulty of PCa treatment.

The initiation, progression, castration resistance, and metastasis of PCa are complex and comprehensive processes in which various types of immune cells play a crucial role. It has been reported that CD4^+^ T cells, including multiple T helper (Th) cell types, are involved in PCa progression. Th1 cells inhibit the growth of PCa by producing Th1-type cytokines, such as IFN-γ and IL-2, to activate CD8^+^ T cell- and NK cell-mediated cytolytic functions 3. In addition, Treg cells and Th2 cells are regarded as protumor effector cells, which may be related to the immunosuppressive factors they secrete, such as TGF-β and IL-10 4, 5. Tumour-infiltrating B cells play an important role in the progression, tumour recurrence and therapy resistance of PCa 6. The functions of tumour-associated macrophages (TAMs) and tumour-associated neutrophils (TANs) act as a “double-edged sword” based on functional polarization in PCa. M1-type TAMs and N1-type TANs play antitumor roles by promoting the inflammatory response 7, 8. In contrast, M2-type TAMs and N2-type TANs promote tumour growth and angiogenesis, tissue remodelling, and metastasis 9, 10. As cytotoxic lymphocytes, NK cells include two major subpopulations in humans, CD56^bright^ and CD56^dim^ cells 11. These two types of NK cells acquire greater cytolytic capacity by producing a variety of proinflammatory cytokines (including IFN-γ and TNF-α) and exhibiting greater cytotoxicity 12. In addition, myeloid-derived suppressor cells (MDSCs) 13, dendritic cells (DCs) 14, and macrophages 15 are also thought to be associated with the PCa progression.

Regulation of immune cells, including reactivating antitumor immune cells or suppressing protumor immune cells, is considered a promising treatment for PCa 6. Cytotoxic lymphocyte antigen-4 (CTLA-4) and programmed cell death protein 1 (PD-1) are expressed on Treg and naïve T cells. Tumour cells escape immunity by binding to CTLA-4 or PD-1 to promote the differentiation and activity of Treg cells and inhibit the proliferation and function of effector T cells 16, 17. Several preclinical studies and clinical trials targeting CTLA-4 and PD-1 are ongoing in PCa 18. That research is exciting, but many PCa patients do not respond to treatment with mAbs against PD1 or CTLA-4. The reason may be that tumour cells achieve immune escape through other pathways 19. Therefore, it is urgent and crucial to explore new molecules with immunomodulatory functions for the treatment of PCa.

SEPT5 belongs to the SEPTIN (SEPT) family, which is a conserved family of 13 GTP-binding proteins that were originally discovered in yeast and are described as a group of cell division cycle-regulating genes 20, 21. The SEPT family has a wide range of biological functions in eukaryotes, including cell division, determination and maintenance of cell polarity, microtubule and actin function, vesicle trafficking, exocytosis, and apoptosis 22. Regulation of cell migration, including immune cell migration, is one of the most important functions of the SEPT family, which suggests that the SEPT family may play an immunomodulatory role. In recent years, the functions of SEPT2, SEPT4, SEPT7 and SEPT9 in tumours have been successively reported 23-27, revealing the importance of the SEPT family in the pathogenesis of tumours.

SEPT5 is abundantly expressed mainly in mouse and human brains and thus is involved in the pathogenesis of neurological diseases, such as Parkinson's syndrome, Alzheimer's disease, and autoimmune cerebellar ataxia 28-30. SEPT5 is frequently found in haematologic malignancies as a partner gene with the MLL gene 31, 32. SEPT5 is rarely reported in solid tumours. Coincidentally, SEPT5 staining was observed in 10/19 (52.6%) PCa patients by Capurso 33. However, the difference in SEPT5 expression between tumour tissue and normal tissue and the function of SEPT5 in PCa are unclear.

In the present study, we explored the expression patterns of SEPT5 in tumours, especially in PCa. The prognostic values of SEPT5 in PCa were then evaluated. In addition, we investigated the correlation between SEPT5 expression and immune infiltration levels, and the function of SEPT5 in PCa progression and immune infiltration was verified in a mouse allograft animal model. Furthermore, the correlation between SEPT5 expression and cytokine-related gene expression was evaluated in PCa.

## Materials and methods

### Data source, SEPT5 expression and survival analysis

The gene expression profiles and the corresponding clinical information of PCa patients were downloaded from The Cancer Genome Atlas (TCGA) database, which included 499 PCa samples and 52 adjacent normal tissues. In addition, the GSE6956 (including 20 normal samples and 69 PCa samples) and GSE55945 (including 8 normal samples and 13 PCa samples) datasets were extracted from the GEO database. The expression of SEPT5 was analyzed after normalization of the raw data using R Studio. SEPT5 expression in PCa samples with different Gleason scores was analyzed using the UALCAN database (http://ualcan.path.uab.edu/index.html). The correlation between SEPT5 expression and survival in PCa was analyzed using the cBioPortal database (www.cbioportal.org/) based on the MSKCC dataset (Cancer Cell, 2010) and TCGA dataset (Firehose Legacy).

### Immune infiltration analysis

The TISIDB database (http://cis.hku.hk/TISIDB/index.php) is an integrated repository portal for tumour-immune system interactions based on TCGA datasets 34 and was used to evaluate the relationship between SEPT5 expression and 28 immune infiltrates in PCa, including B cells, CD8^+^ T cells, CD4^+^ T cells, dendritic cells, neutrophils, and macrophages. Then, the data on immune cell abundance were downloaded, and 498 prostate cancer samples were divided into two groups according to the expression of SEPT5: low SEPT5 expression and high SEPT5 expression. The abundance of immune cells in the two groups of samples was then recalculated.

### Correlation analysis

The correlation between SEPT5 expression and the expression of various types of chemokine-related genes and the correlation between SEPT5 expression and the expression of various types of cytokine-related genes in PCa were both assessed using the cBioPortal database based on the DKFZ dataset (Cancer Cell, 2018) and the Tumour Immune Estimation Resource database (Timer, http://timer.comp-genomics.org/), respectively.

### Cell lines and cell culture

The human PCa cell lines PC3, DU145, 22RV1, and LNCaP and the murine PCa cell line RM1 were purchased from the American Type and Culture Collection (ATCC, Manassas, VA) and cultured in RPMI 1640 medium containing 10% fetal bovine serum, 100 U/ml penicillin and 100 μg/ml streptomycin. The human prostate epithelial cell line RWPE1 was cultured using Defined Keratinocyte SFM (10744019, Gibco). All cells were maintained at 37 °C in a 5% CO_2_ incubator.

### Gene silencing

Crispr sgRNAs were designed in chopchop (http://chopchop.cbu.uib.no/#) and synthesized by Thermo Fisher Scientific. The sgRNA sequences for mouse cell lines were as follows: sgRNA1: TTTCGACTTCACGCTCATGG; sgRNA2: TGACCGACCTGTATAAGGACCGG. The sgRNA sequences for human cell lines were as follows: sgRNA3: ACGCTGTCAACAACACCGAGTGG; sgRNA4: GAAGCCCATCACCGACTATGTGG. The lentiCRISPR v2 lentivirus was purchased from Addgene. Cells were transfected with sgRNAs using Lipofectamine LTX (Invitrogen) followed by geneticin selection. Knockdown was confirmed by western blotting and real-time PCR.

### Western blotting analysis

Cell samples were lysed in RIPA buffer (Beyotime) containing protease and phosphatase inhibitors (Thermo Fisher Scientific) and then centrifuged to separate the total protein in the supernatant. Equal amounts of total protein were analyzed via protein electrophoresis using a 12% SDS polyacrylamide gel. After electrophoresis, the proteins were transferred to polyvinylidene difluoride (PVDF) membranes. Then, the PVDF membranes were blocked with 5% nonfat milk and incubated with dedicated primary antibodies (listed in Table S1). After incubation overnight at 4 ℃, the membranes were incubated with the corresponding horseradish peroxidase conjugated (HRP)-conjugated secondary antibodies for 1.5 hours at room temperature. Samples were developed using chemiluminescence substrate (Millipore) and exposed via a chemiluminescence/fluorescence/condensation gel imaging analysis system (Beijing Saizhi Entrepreneur Technology Co., Ltd). The proteins were quantified using ImageJ software.

### RNA extraction and quantitative real-time PCR analysis

Total RNA was extracted using TRIzol reagent (Thermo Fisher), and a high-capacity cDNA reverse transcription kit (Thermo Fisher) was used for cDNA synthesis. Subsequent qPCR was performed using Taq Pro Universal SYBR qPCR Master Mix (Vazyme) on an ABI 7500 Real-time PCR system (Applied Biosystems). The relative mRNA levels were calculated via the cycle threshold (2^-ΔΔCT^) method and were normalized to actin levels. The primers used are listed in Table S2.

### Animals and animal experimental protocols

Male pathogen-free C57BL/6J mice at 6-7 weeks of age were purchased from Beijing Vital River Laboratory Animal Technology Co., Ltd. and maintained in the SUSTech Laboratory Animal Research Center. The animal protocol was approved by the Institutional Animal Ethics Committee of Southern University of Science and Technology (SUSTech-JY2020140).

The mice were sorted into three groups based on their weight after acclimatization for 1 week in the animal research center, and 8 mice per group were used in the experiments. Then, 5 × 10^5^ RM1 cells with SEPT5 knockdown or corresponding control cells were subcutaneously injected into the right flanks of the indicated mice. The body weight of the mice and tumour size were monitored twice a week, and the tumour volume was calculated. A tumour size exceeding 1000 mm^3^ was used as the endpoint of the experiment.

At the endpoint of the experiment, the mice were sacrificed, and the tumours were removed and weighed. Subcutaneous tumours were isolated and divided into 2 parts aseptically. One half of the tumour was shredded and made into a single-cell suspension using a Tumour Dissociation Kit (Miltenyi) for mass cytometry (CyTOF) detection. The other halves were kept in formalin at 4 °C.

### Tissue microarray and immunohistochemistry (IHC) detection

Human PCa tissue microarrays (HProAde045PG01) were purchased from Outdo Biotech Co., Ltd. and used to evaluate the expression of SEPT5 in PCa patients. The dataset contained 3 normal prostate tissues and 42 PCa tissues. Human PCa tissue microarrays (M079Pr01) were purchased from Bioaitech Co., Ltd. and used to evaluate the expression of SEPT5 and CD8 in PCa patients. The dataset contained 9 normal prostate tissues and 70 PCa tissues.

For IHC detection, the tissue microarray slides and mouse tumour tissue slides from animal experiments were deparaffinized and hydrated. Heat-induced antigen retrieval (HIER) was carried out for all sections in citrate buffer (0.01 M, pH 6.0) (Beyotime) using a microwave. Blocking of endogenous peroxidase was performed by incubation with endogenous peroxidase blocking buffer (Beyotime). Then, the slides were blocked with normal goat serum (Panera) and incubated with the indicated primary antibodies (listed in Table. S1) at 4 °C overnight. An ElivisionTM Plus Polymer HRP IHC Kit (Biotechnologies) was used for subsequent detection. After primary antibody incubation, slides were incubated with secondary antibody supplied by the kits described above for 20 minutes at room temperature, and then, the colour reaction was developed with DAB solution. Finally, the sections were counterstained with haematoxylin, dehydrated, coverslipped and visualized with a Digital Pathology Scanner (Leica Microsystems). The SEPT5 staining score was calculated based on the intensity and percentage of staining. The Ki67-positive cells, CD8-positive cells and granzyme A (GZMA)-positive cells were counted using ImageJ.

### Immune cell detection via CyTOF

Immune cells were detected via CyTOF according to the manufacturer's manual. In brief, cells in a single cell suspension from tumour tissues were counted, and 3 × 10^6^ were first incubated with cisplatin (Fluidigm Sciences). For staining, cells were blocked with 50 µl CD16/32 antibody (BD Biosciences) for 15 minutes at room temperature, and then, 50 µl CyFACS buffer containing a surface antibody cocktail was added directly to the blocking solution and incubated with cells for 30 minutes at room temperature. The metal-labelled antibodies are listed in Table S1. Next, the cells were successively fixed with 1.6% paraformaldehyde (Fluidigm Sciences) and incubated with intercalator-Ir (Fluidigm Sciences). After incubation overnight at 4 ℃, the cells were washed twice and resuspended in 1 × EQ Four Element Calibration Beads (Fluidigm Sciences). Finally, 300,000 events were collected on a CyTOF mass cytometer (Fluidigm Sciences). The data were analysed on the Cytobank website after normalization.

### RNA-seq analysis

RNA-seq analysis was performed by Gene Denovo Biotechnology Co. In brief, the SEPT5 knockdown cells and corresponding vector cells on RM1 cell lines were harvested directly using 1 ml of TRIzol reagent per 10 cm plate to isolate the total RNA (three biological replicates for each group). RNA quality was assessed and checked. Then, eukaryotic mRNA was enriched by Oligo(dT) beads, and the enriched mRNA was fragmented into short fragments using fragmentation buffer and reverse transcribed into cDNA. The resulting cDNA library was sequenced using an Illumina Novaseq6000 system. RNA differential expression analysis between the SEPT5 knockdown group and the SEPT5 vector group was performed using DESeq2 software. A fold change value of 2.0 and a p value smaller than 0.01 were used as thresholds to distinguish dysregulated genes. Additionally, DO, GO, KEGG, and Reactome enrichment analyses were performed.

### Mouse Chemokine Array

The chemokines levels in cell supernatant were detected using Raybiotech kits (GSM-CHE-1) according to the standard operating procedures of Raybiotech. This chemokine Array contain 25 types of chemokines. In brief, the cell supernatant from SEPT5 knockdown cells and corresponding control cells were isolated firstly. For detection, the Chemokine Array were Incubated with 100 ul cell supernatant at 4 ℃ overnight after blocking. Then, removing the cell supernatant, the Chemokine Array were Incubated with biotin conjugated anti-Cytokines for 2 hours at room temperature after wasing. Next, removing the biotin conjugated anti-Cytokines, Cy3-Streptavidin were added to the Chemokine Array for 1 hours at room temperature after washing. Finally, the Chemokine Array were washed, spun dry, and canned with an Agilent SureScan Dx Microarray Scanner at 532 nm, Power 100%.

### Statistical analysis

All data in this study are presented as the means ± SEM. Statistical analysis was performed using R (3.6.1) for the datasets from TCGA and the GEO database. Statistically significant differences between two groups were assessed using an independent-sample t test, and comparisons between more than two groups were achieved with one-way ANOVA followed by LSD or Dunnett's T3 test using SPSS version 20.0. Spearman correlation was used to analyze correlations between two indicators. P < 0.05 was considered statistically significant, and all statistical analyses were performed using GraphPad Prism, Microsoft Excel and R software.

## Results

### The expression of SEPT5 was elevated and associated with poor prognosis in PCa

**Figure 1:**
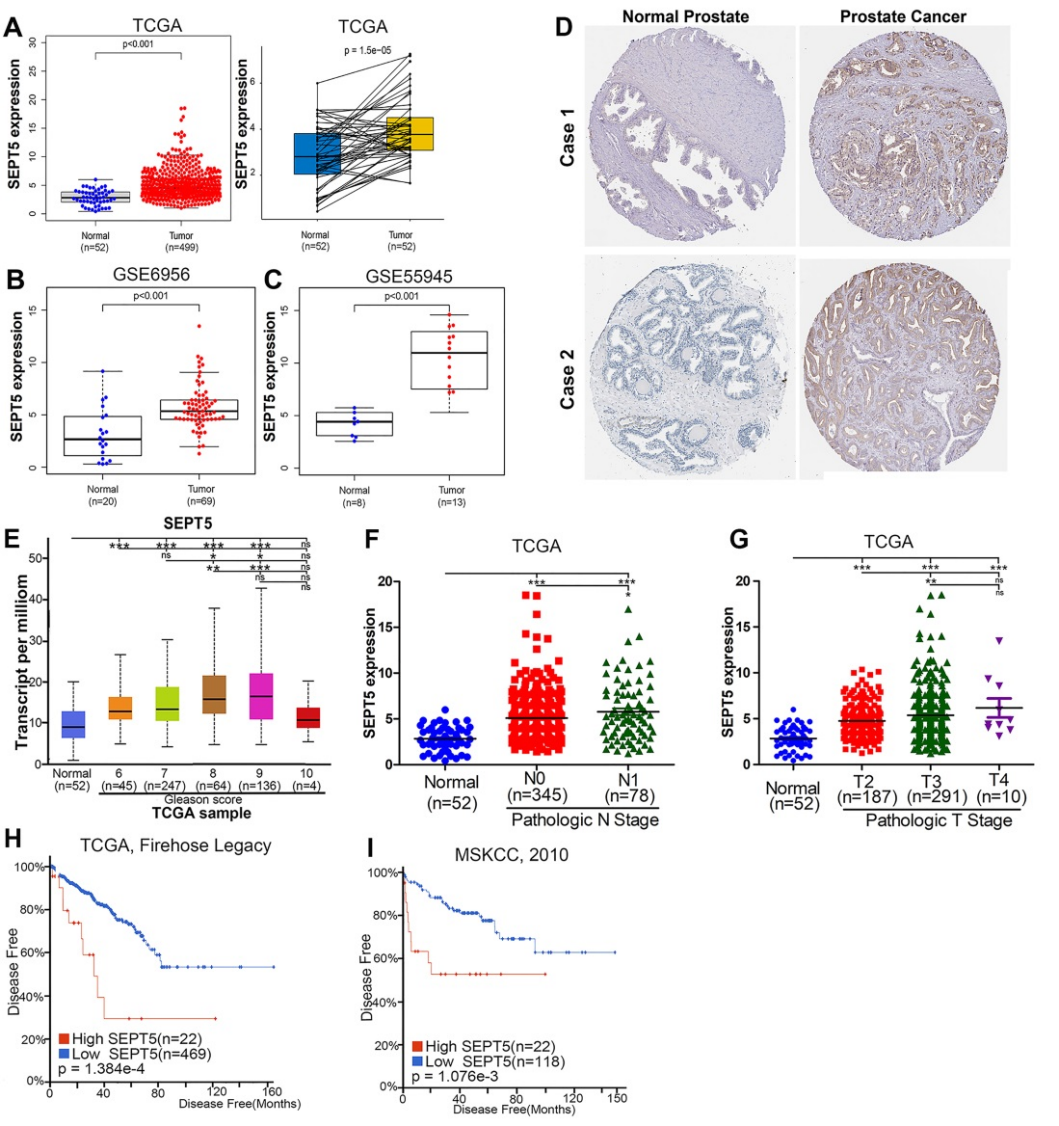
** The expression of SEPT5 was elevated and associated with poor prognosis in PCa based on public database.** Comparison of SEPT5 gene expression between PCa tissues and normal tissues in a TCGA dataset (A), GSE6956 dataset (B), and GSE55945 dataset (C); (D) Comparison of SEPT5 protein expression between PCa tissues and normal prostate tissue based on the IHC staining results from the Human Protein Atlas project; SEPT5 expression compared with Gleason score (E), N stage (F) and T stage (G) in the TCGA dataset; Kaplan-Meier plot for disease-free survival of patients in TCGA dataset (H) and MSKCC (I) datasets.

To analyze the role of SEPT5 in cancer, we first explored the expression of SEPT5 based on TCGA datasets. The results revealed a discrepancy in SEPT5 expression between tumour tissues and corresponding normal tissues in multiple cancer types (Fig. S1). In detail, there was lower SEPT5 expression in glioblastoma multiforme, head and neck squamous cell carcinoma, kidney chromophobe, stomach adenocarcinoma, and uterine corpus endometrial carcinoma than in normal tissues. In addition, compared to normal tissues, higher SEPT5 expression was observed in breast invasive carcinoma, cholangiocarcinoma, liver hepatocellular carcinoma, and lung squamous cell carcinoma, among others. In terms of PCa, the expression of SEPT5 was higher in 499 PCa tumour tissues than in 52 normal prostate tissues (*p* < 0.001) (Fig. 1A). We also analyzed SEPT5 expression in 52 pairs of PCa samples and matched adjacent normal samples in the TCGA dataset, and the expression of SEPT5 was still higher in the cancer samples than in the matched adjacent normal samples (*p* < 0.01) (Fig. 1A). Furthermore, higher SEPT5 expression was found in other PCa datasets (*p* < 0.01) (Fig. 1B, C). Meanwhile, we also found that the protein expression of SEPT5 was stronger in PCa tissues than in normal prostate tissues based on IHC staining results from the Human Protein Atlas project (Fig. 1D).

Excitingly, higher SEPT5 expression appeared to be associated with poor prognosis in PCa. Firstly, SEPT5 expression appeared to be correlated with Gleason grade in PCa patients, the expression level of SEPT5 was elevated with increasing Gleason score in TCGA datasets (Fig. 1E). In addition, we also explored the relationship between SEPT5 expression and pathologic stage in PCa specimens based on TCGA datasets, and this analysis revealed that higher expression of SEPT5 was associated with higher N stage (Fig. 1F) and T stage (Fig. 1G). Additionally, we found that higher SEPT5 expression was associated with shorter DFS of PCa patients in TCGA (*p* < 0.01) (Fig. H) and MSKCC (*p* < 0.01) datasets (Fig. I).

**Figure 2:**
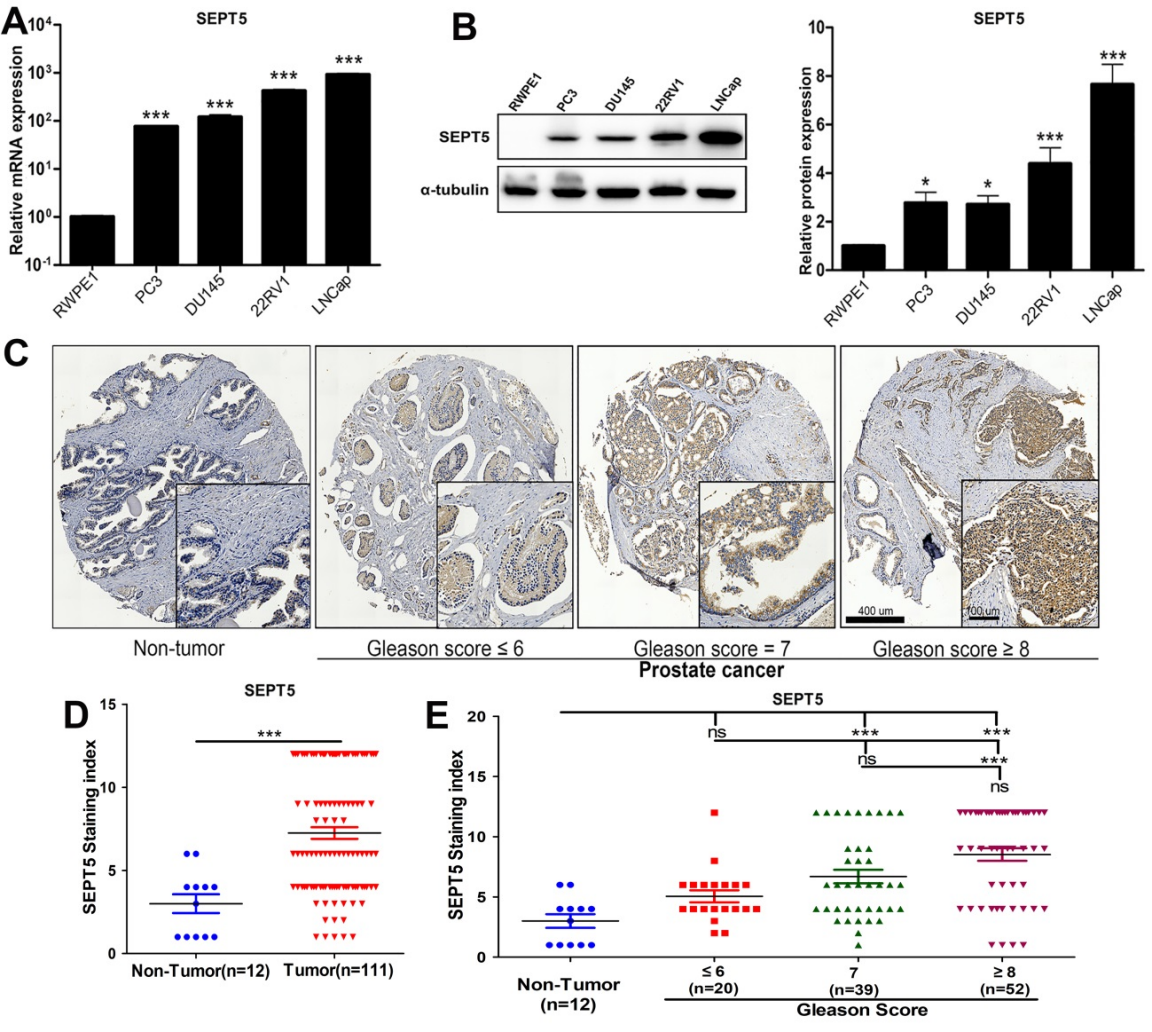
** The expression of SEPT5 was increased in PCa cell lines and associated with gleason score of PCa samples.** (A) Transcript level of SEPT5 in human PCa cell lines and prostate epithelial cells was detected; (B) Western blotting results showing SEPT5 expression in human PCa cell lines and prostate epithelial cells; (C) The expression levels of SEPT5 in normal prostate tissues and PCa tissues were detected by IHC; (D) Quantitative analysis of the SEPT5 expression in normal prostate tissues and PCa tissues; (E) Quantitative analysis of the SEPT5 expression in normal prostate tissues and PCa tissues with different Gleason Score. Data are depicted as the mean ± SEM. * P < 0.05, ** P < 0.01 and *** P < 0.001, determined by one-way ANOVA. n.s., not significant.

Next, the results obtained in public databases were validated. Firstly, we detected the mRNA expression level of SEPT5 in PCa cell lines, and found that the mRNA level of SEPT5 was more than 10-fold higher in all PCa cell lines than in RWPE1 cells (*p* < 0.001), especially in 22RV1 and LNCaP cells (Fig. 2A). Then, extreme upregulation (more than two fold, *p* < 0.05) of SEPT5 expression was observed in all of the PCa cell lines compared with RWPE1 cells (Fig. 2B). Meanwhile, we examined the expression of SEPT5 in PCa tissues and normal prostate tissue using the tissue array, and the results demonstrated higher SEPT5 expression in PCa tissues (*p* < 0.001) (Fig. 2D). Excitingly, the mean staining intensity of SEPT5 gradually increased with increasing Gleason score (Fig. 2C and E).

### SEPT5 expression was associated with immune cell infiltration in the tumour microenvironment (TME) of PCa

**Figure 3:**
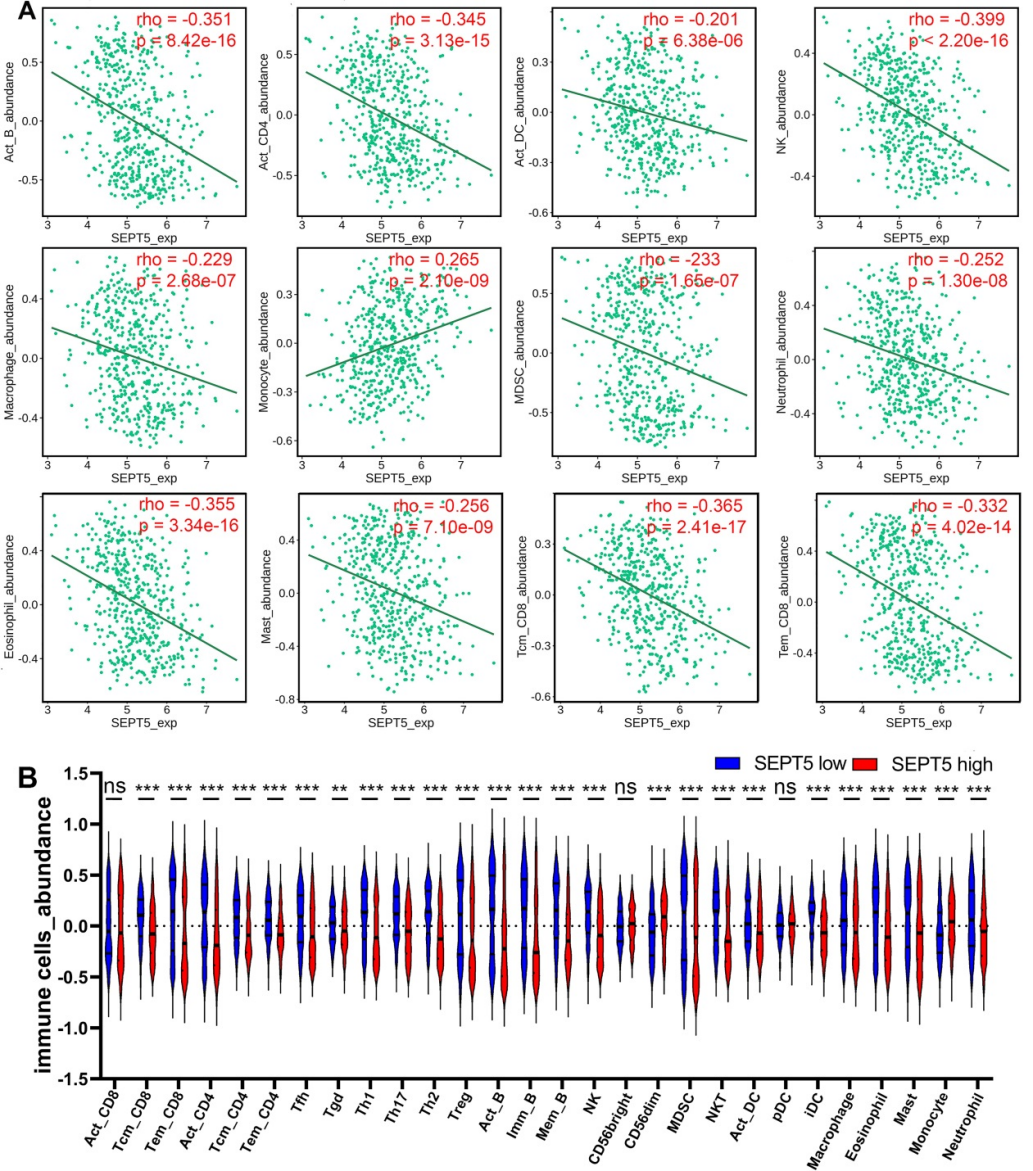
** SEPT5 expression were associated with major immune infiltration levels in PCa based on the TCGA dataset.** (A) SEPT5 expression is significantly correlated with infiltration levels of activated B cells, activated CD4^+^ T cells, activated DCs, NK cells, macrophages, monocytes, MDSCs, neutrophils, eosinophils, mast cells, central memory CD8^+^ T cells, and effector memory CD8^+^ T cells; (B) Violin plot showed the significant differences in the abundance of major immune cells between the high SEPT5 group and the low SEPT5 group. Data are depicted as the mean ± SEM. * P < 0.05, ** P < 0.01 and *** P < 0.001, determined by one-way ANOVA. n.s., not significant.

Next, we investigated whether SEPT5 can play an immunomodulatory role in PCa. We first investigated the relationship between SEPT5 expression and the infiltration levels of 28 types of immune cells in 30 cancer types from the TISIDB database. The results showed that SEPT5 expression had significant correlations with immune infiltration levels in many types of cancer, such as glioblastoma multiforme, breast invasive carcinoma, and lung squamous cell carcinoma (Fig. S2). We focused on PCa and found that SEPT5 expression has significant correlations (*p* < 0.05) with 25 types of immune cell infiltration levels in PCa. In detail, SEPT5 expression was only positively correlated with CD56^dim^ natural killer cell and monocyte infiltration in PCa. Otherwise, SEPT5 expression had significant negative correlations with the other 23 types of immune cell infiltration levels, such as activated CD4^+^ T cells, activated B cells, central memory CD8^+^ T cells, and effector memory CD8^+^ T cells (Fig. 3 and Table 1). Furthermore, we also evaluated the abundance of major immune cells in the low SEPT5 and high SEPT5 expression groups. The results demonstrated that the abundance of monocytes in the high SEPT5 group was higher than that in the low SEPT5 group (*p* < 0.001), while the high SEPT5 group had a lower abundance of activated CD4^+^ T cells, activated B cells, central memory CD8^+^ T cells, and effector memory CD8^+^ T cells, among others (Fig. 3 and and Table S3). Altogether, these results suggest that SEPT5 may play an important role in immune cell infiltration in PCa.

### Downregulation of SEPT5 repressed the growth of PCa cells* in vivo*

**Figure 4:**
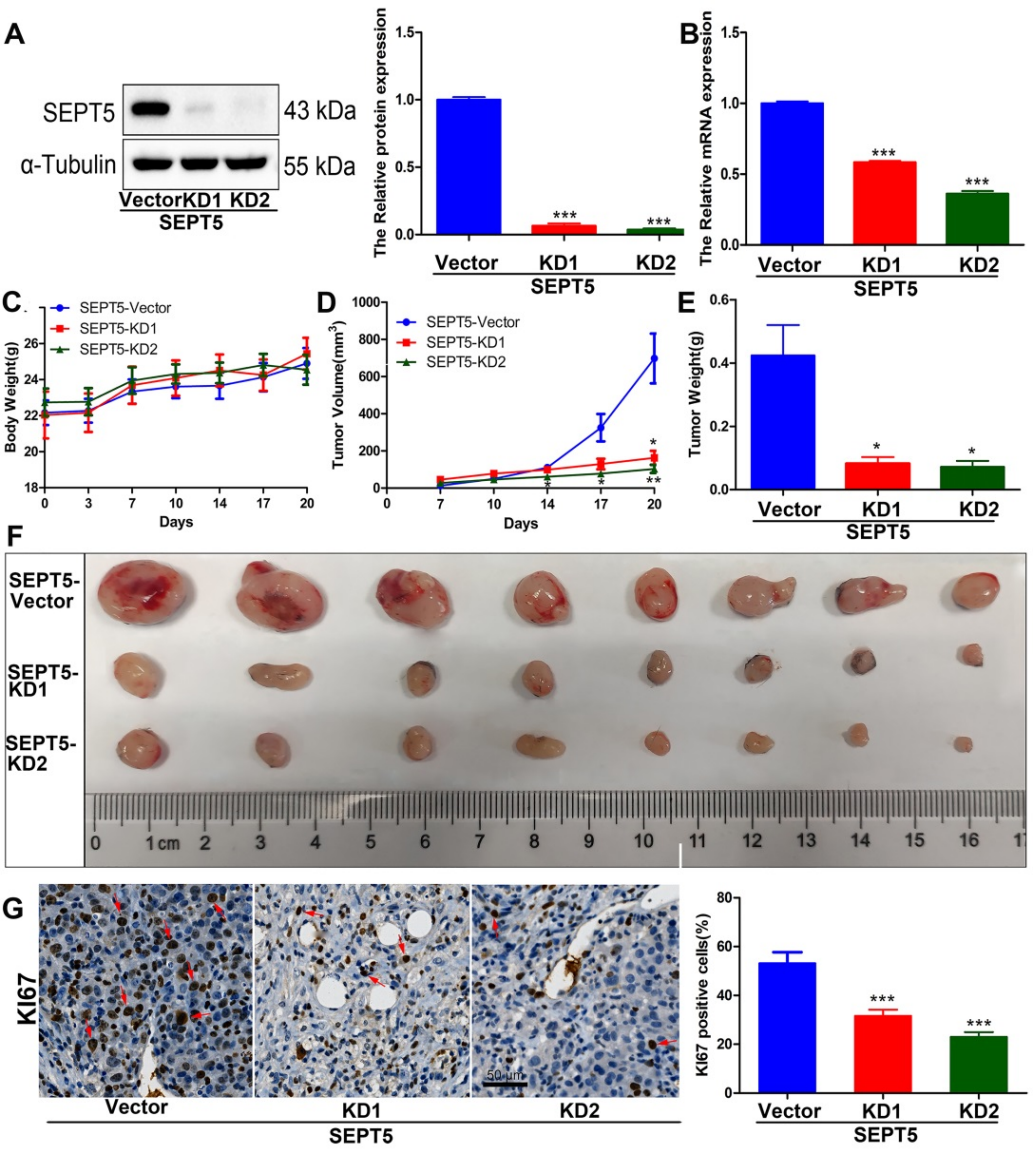
** Downregulation of SEPT5 repressed the growth of PCa cells *in vivo.*** Knockdown of SEPT5 was verified by western blotting (A) and real-time PCR (B); Then, a mouse allograft animal model was established, and mouse body weight (C) and tumour volume (D) were monitored; At the endpoint of the experiment, the tumour weight was measured (E), and all tumours were photographed (F); The expression of Ki67 was detected by IHC, and a representative image and statistical results are presented (G). Data are depicted as the mean ± SEM. * P < 0.05, ** P < 0.01 and *** P < 0.001, determined by one-way ANOVA.

To evaluate whether SEPT5 regulates the growth of PCa cells by regulating immune cells. We specifically knocked down SEPT5 via CRISPR-Cas9 in RM1 cells, and knockdown was confirmed by western blotting (Fig. 4A) and real time PCR (Fig. 4B). Then, the regulatory effect of SEPT5 on PCa cell growth was evaluated in a mouse allograft animal model. The results illustrated that the weight of mice in the SEPT5 knockdown group did not change significantly compared with that in the control group (*p* > 0.05) (Fig. 4C). Compared with the control group, the tumour size (Fig. 4D) and tumour weight (*p* < 0.01) (Fig. 4E and F) in the SEPT5 knockdown group were significantly lower. In addition, Ki67, a proliferation marker, was used to evaluate proliferation ability. We found that the Ki67-positive cells of tumour tissues was significantly lower in the SEPT5 knockdown group compared with the control group (*p* < 0.01) (Fig. 4G). To summarize, downregulation of SEPT5 inhibited the growth of PCa cells *in vivo*.

### The population and composition of immune cells in tumour tissue were altered by downregulation of SEPT5

**Figure 5:**
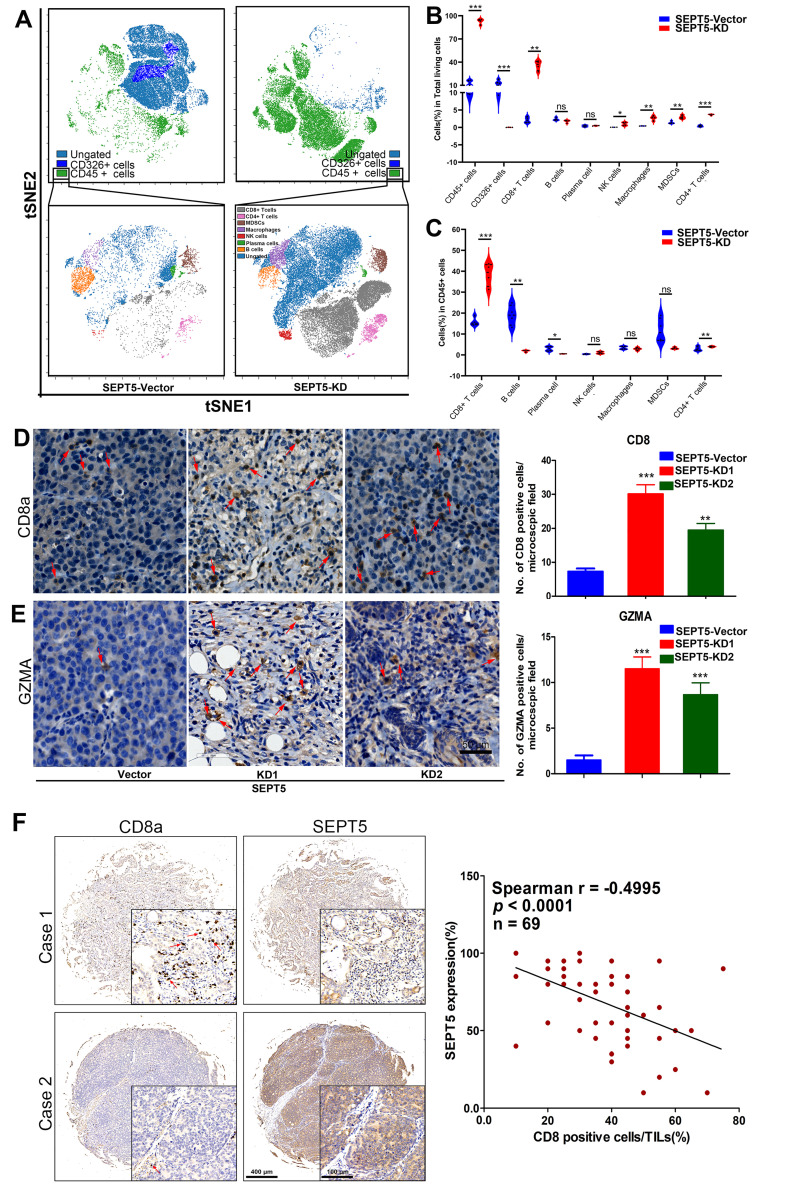
** Downregulation of SEPT5 altered the population and composition of immune cells.** The population of immune cells was detected via CyTOF, (A) A representative images of the cell subsets in living cells and in CD45-positive cells; (B) The proportion of cell subsets in living cells was counted (n = 4); (C) The proportion of cell subsets in CD45-positive cells was counted (n = 4); The expression of CD8a and GZMA was detected by IHC, (D) A representative image and statistical results for CD8a; (E) A representative image and statistical results for GZMA; (F) The correlation between SEPT5 expression and CD8^+^ T cell infiltration in PCa patients; Data are depicted as the mean ± SEM. * P < 0.05, ** P < 0.01 and *** P < 0.001, determined by one-way ANOVA.

To determine the effect of SEPT5 on immune cells in PCa, we detected the major immune cells via CyToF. First, we found that the proportion of CD45-positive cells in the tumour tissue increased from 13.05% to 92.82% after SEPT5 knockdown (*p* < 0.001). In addition, the proportions of CD8^+^ T cells, CD4^+^ T cells, MDSCs, NK cells and macrophages were also increased in the tumour tissue in the SEPT5 knockdown group compared with the control group (*p* < 0.05) (Fig. 5A and B, Table 2). Next, we focused on the composition of immune cells. Compared with the control group, the proportion of CD4^+^ T cells and CD8^+^ T cells was significantly increased (*p* < 0.001) while the proportion of B cells and plasma cells was decreased among the CD45-positive cells in the SEPT5 knockdown group (*p* < 0.05) (Fig. 5A and C, Table 2).

We found that CD8^+^ T cells were the most important immune cell type in PCa, and the proportion of CD8^+^ T cells increased among both total cells and CD45-positive cells (more than 16- and 2-fold, respectively, *p* < 0.001) in the SEPT5 knockdown group. Therefore, we focused on the function of SEPT5 on CD8^+^ T cells. Next, we verified the CyTOF results using IHC and determined that CD8^+^ T cells obviously increased after SEPT5 knockdown (Fig. 5D). Meanwhile, we detected the expression of GZMA. GZMA expression was rarely detected in tumour tissues in the SEPT5-vector group, and GZMA expression in the SEPT5-KD1 and SEPT5-KD2 groups increased approximately 8-fold and 6-fold, respectively (Fig. 5E). Subsequently, we analyzed the correlation between SEPT5 expression and CD8^+^ T cell infiltration in clinical samples, and the results showed that the proportion of CD8^+^ cells was lower in the PCa samples with high SEPT5 expression, CD8^+^ T cell infiltration was significantly negatively correlated with the expression of SEPT5 in PCa tissues (*p* < 0.001, Fig. 5F).

### 3.5 Correlation analysis between SEPT5 expression and cytokine gene expression in PCa

**Figure 6:**
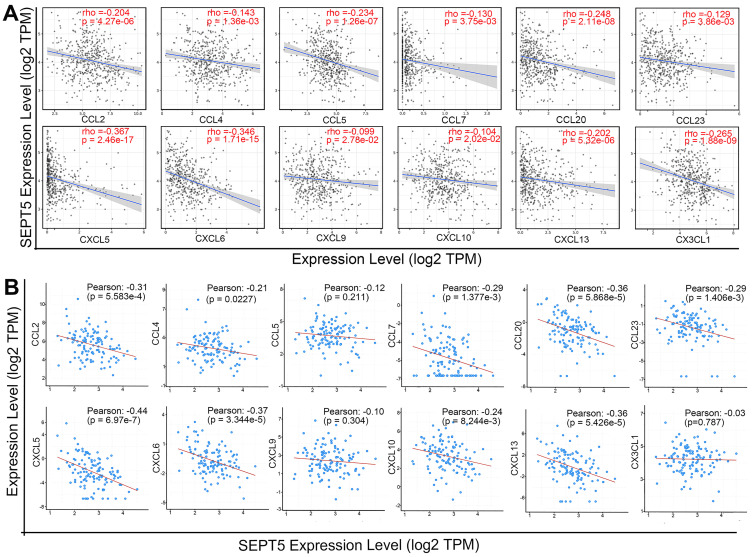
** SEPT5 expression was negatively correlated with chemokine gene expression related to CD8^+^ T cell infiltration.** The correlation between SEPT5 expression and chemokine gene expression related to CD8^+^ T cell infiltration was analyzed based on the TCGA dataset (A) and DKFZ dataset (B).

After confirming that the proportion of some types of immune cells, especially CD8^+^ T cells, in the tumour tissue of the SEPT5 knockdown group were increased, we explored the mechanism by which immune cells increased after SEPT5 knockdown. Therefore, we first performed correlation analysis between SEPT5 expression and the expression of 38 chemokine genes in PCa based on the TCGA dataset (n = 498) (Table S4). The results showed that the expression of 26 of the 38 chemokines was significantly correlated with SEPT5 expression (*p* < 0.05). We focused on chemokines associated with CD8^+^ cell recruitment and found that the expression levels of CCL2, CCL4, CCL5, CCL7, CCL20, CCL23, CXCL5, CXCL6, CXCL9, CXCL10, CXCL13, and CX3CL1 were significantly negatively correlated with SEPT5 expression in PCa (Fig. 6A). Next, we validated these results using the DKFZ dataset (n = 324) and found that the expression of most of these chemokines was also negatively correlated with SEPT5 expression in the DKFZ dataset (Fig. 6B).

**Figure 7:**
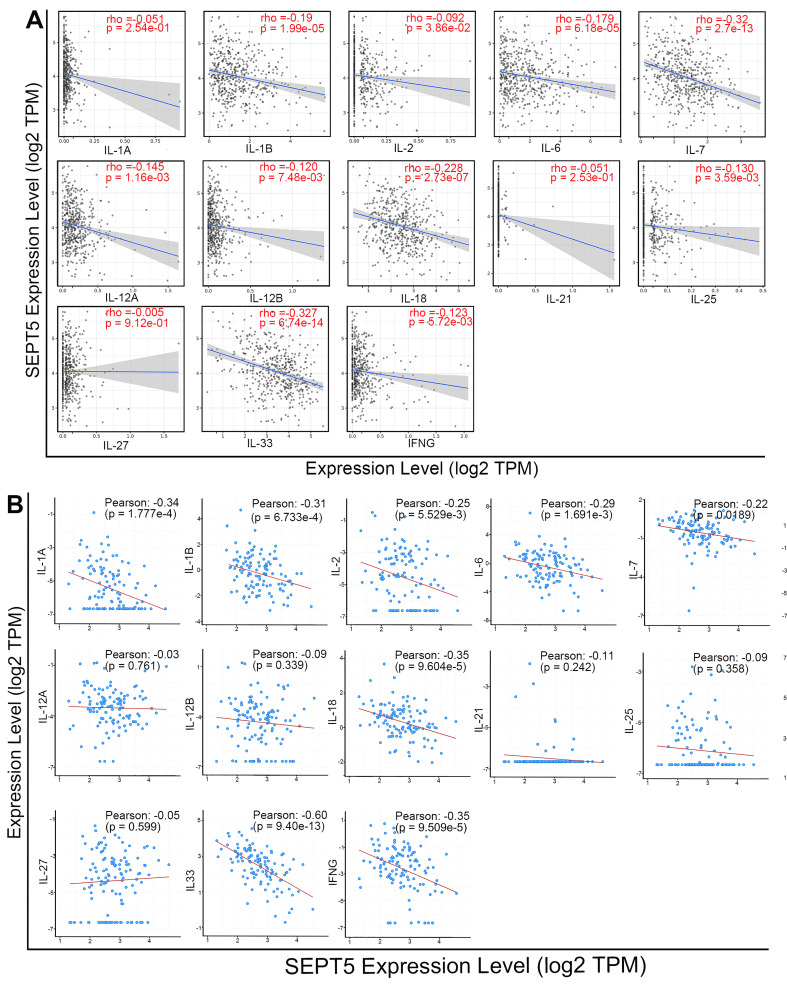
** SEPT5 expression was negatively correlated with cytokine gene expression related to CD8^+^ T cell activation.** The correlation between SEPT5 expression and cytokine gene expression related to CD8^+^ T cell activation was analyzed based on the TCGA dataset (A) and DKFZ dataset (B).

Meanwhile, we analyzed the correlation between SEPT5 expression and cytokine gene expression in the TCGA dataset, since cytokines can regulate the activation and differentiation of immune cells. Among the 48 cytokines we investigated, 2 were positively correlated with SEPT5 expression, and 26 were negatively correlated with SEPT5 expression (*p* < 0.05; Table S5). The correlation between the expression of 13 cytokine genes related to CD8^+^ T cell activation and SEPT5 expression was analyzed (Fig. 7A); Among them, the expression of 10/13 genes was significantly negatively correlated with SEPT5 expression, such as IL-6 and IL-7. These results were also verified using the DKFZ dataset (Fig. 7B).

### 3.6 Downregulation of SEPT5 reactivated cytokine-related signaling pathways and promoted the production of IFNG-inducible chemokines and cytokines expression

**Figure 8:**
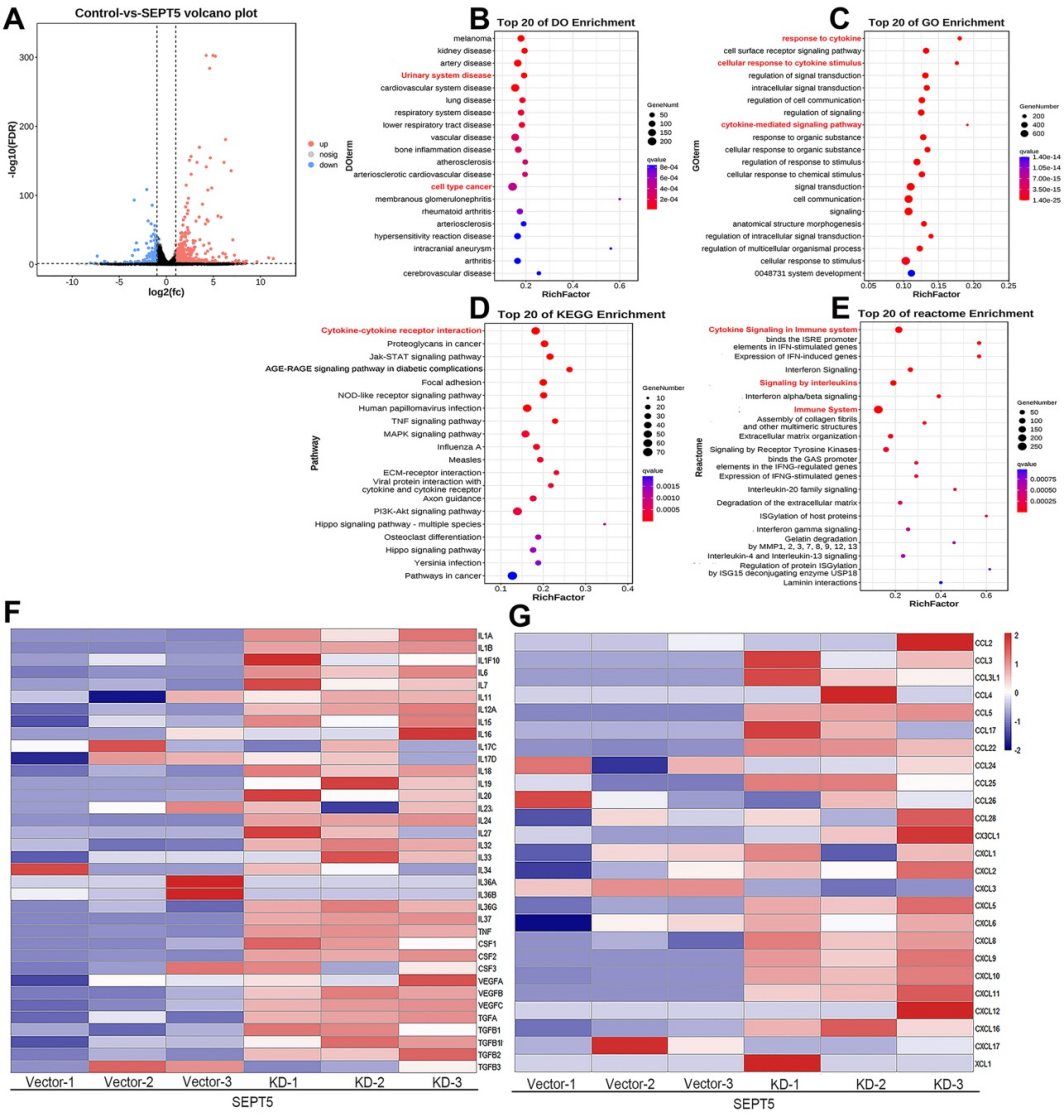
** Downregulation of SEPT5 activated cytokine-related signaling pathways.** RNA-seq was performed in RM1 cells with SEPT5 knockdown and corresponding control cells. (A) The 1444 upregulated and 164 downregulated DEGs are presented; (B) Top 20 DEGs identified by DO enrichment analysis; (C) Top 20 DEGs identified by GO enrichment analysis; (D) Top 20 DEGs identified by DO KEGG analysis; (E) Top 20 DEGs identified by Reactome enrichment analysis; (F) Heatmap showed the expression of cytokine-related genes; (G) Heatmap showed the expression of chemokine-related genes.

In total, 1444 upregulated and 164 downregulated DEGs were revealed based on an analysis of the RNA transcriptomes (Fig. 8A). DO analysis revealed that the increased mRNA in SEPT5 knockdown cells was enriched for diseases such as urinary system disease and cell type cancer (Fig. 8B). Then, the 1444 upregulated and 164 downregulated DEGs were also used for GO, KEGG and reactome enrichment analyses, and the top 20 enrichment results are shown in Fig. 8. C-E, respectively. Cytokine-related pathways were among the top 10 enrichment results in the three enrichment analyses.

Next, an expanded analysis of the gene induction of 36 cytokines, such as those in the IL family and TGF family, in SEPT5-vector and SEPT5-knockdown cells is shown in Fig. 7F. Compared with the control, most of the cytokine expression levels were elevated after SEPT5 knockdown (Fig. 8F, Table. S6), including IL-1A, IL-1B, IL6, IL-7, IL-12A, IL-18, IL-27, and IL-33.

**Figure 9:**
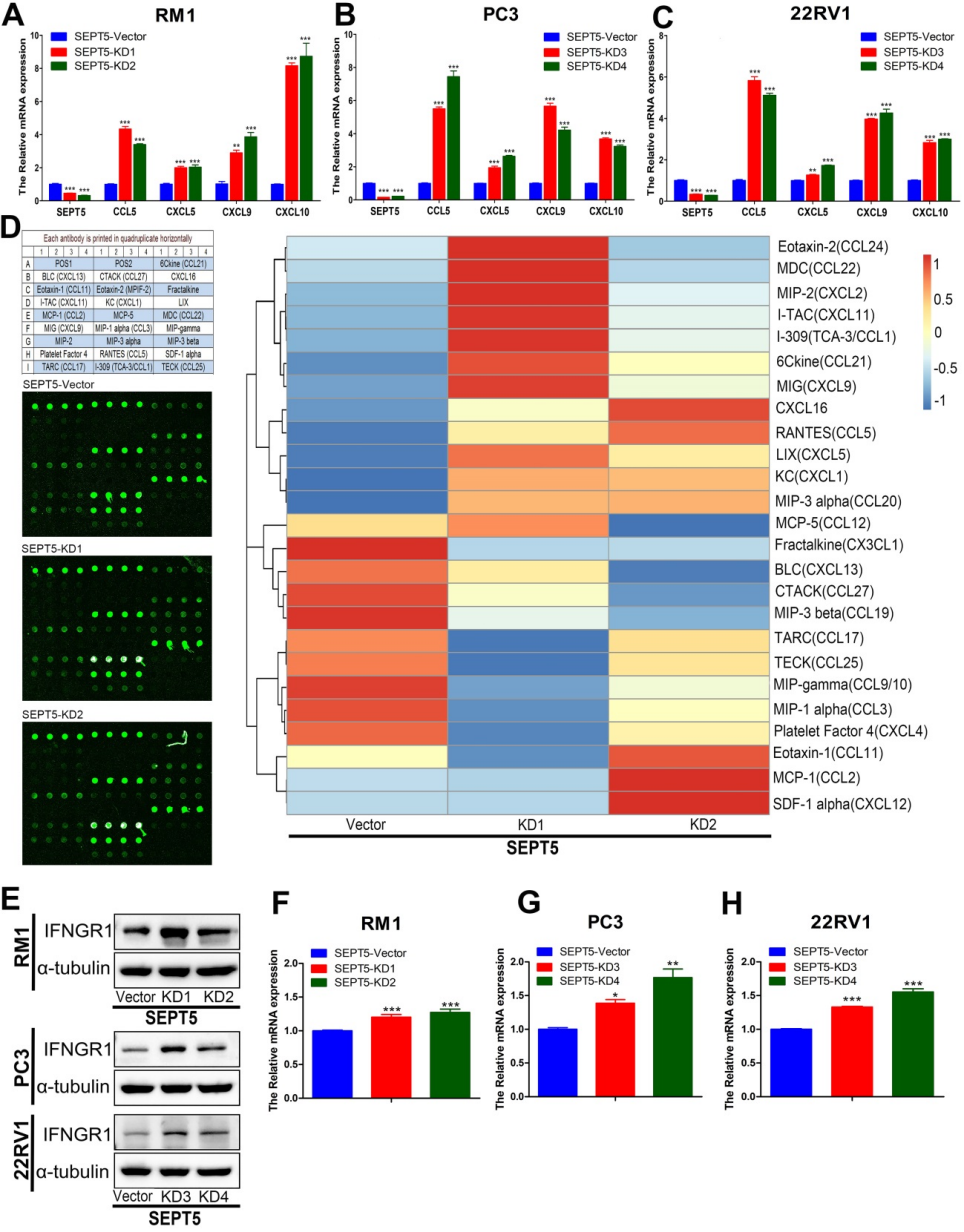
** Downregulation of SEPT5 promoted the expression of IFN-γ-inducible CCL5, CXCL5, CXCL9, CXCL10.** The RNA from PC3, 22RV1 and RM1 cell lines were isolated, and then the expression of SEPT5, CCL5, CXCL5, CXCL9, and CXCL10 at mRNA level was analyzed by real-time PCR in RM1 (A), PC3 (B), and 22RV1 (C) cell lines; (D) The expression of chemokines in RM1 at protein levels were detected using the chemokines array; (E) The expression of IFNGR1 was detect at protein levels in PCa cells; The expression of IFNGR1 was detect at mRNA levels in RM1 (F), PC3 (G), and 22RV1 (H) cell lines. Data are depicted as the mean ± SEM. * P < 0.05, ** P < 0.01 and *** P < 0.001, determined by one-way ANOVA.

In addition, we investigated chemokine expression in SEPT5-vector and SEPT5-knockdown cells based on RNA-Seq results. We found that 12 chemokines were significantly increased (*p* < 0.05) after downregulation of SEPT5, including 4 chemokines associated with CD8^+^ T cell recruitment: CCL5, CXCL5, CXCL9, and CXCL10 (Fig. 8G, Table S7). The increased expression of CCL5, CXCL5, CXCL9, and CXCL10 at mRNA levels in RM1 cells after SEPT5 knockdown were also confirmed by real-time PCR (Fig.9A). Importantly, the expression of those chemokines was consistent in human PCa cell lines (PC3 and 22RV1) as well as RM1 cell line (Fig. 9B-C). The chemokines array also showed that the expression of some kind of the chemokines at protein levels were elevated after SEPT5 knockdown in RM1 cells, include CCL5, CXCL5, CXCL9 and so on (Fig. 9D). Subsequently, to explore the mechanism of increased chemokine levels after down-regulated SEPT5, we observed that SEPT5 expression was significantly negatively correlated with the expression of IFNG in PCa specimens (Fig. S3A), with respect to IFNG receptors, SEPT5 expression was significantly negatively correlated with IFNGR1 expression (Fig.S3B), but not with IFNGR2 expression (Fig.S3C) in PCa specimens based on public database. Finally, we verified the IFNGR1 expression in PCa cell lines with SEPT5 knockdown, and the data showed that the IFNGR1 was also significantly elevated in mRNA and protein levels after SEPT5 knockdown in PCa cells (Fig. 9E-H).

## Discussion

Recently, an increasing number of studies have revealed that the SEPT family is involved in tumorigenesis, including in oral, brain, pancreatic, and melanoma tumours 35. Historically, SEPT9 was the first septin implicated in cancer 36, and SEPT9 may be a new biomarker of colon cancer, head and neck squamous cell carcinoma, and cervical cancer 37-39. However, the role of SEPT5 in cancer, including PCa, has not received enough attention. In this study, we demonstrated that there were significant differences between tumour tissues and corresponding normal tissues in 15 cancer types, including PCa, which indicates that SEPT5 may be involved in tumour pathogenesis. Interestingly, diversification in different tumours showed inconsistent trends in SEPT5 expression. Lower SEPT5 expression was observed in glioblastoma multiforme, which was confirmed by DU 40 using IHC detection and western blotting. In PCa, we found higher SEPT5 mRNA and protein expression in tumour tissue based on bioinformatics analysis. Then, high mRNA and protein expression of SEPT5 was verified in tumour cell lines by western blotting and qPCR. In addition, the protein expression of SEPT5 in PCa specimens and normal prostate specimens was detected using a PCa tissue array, and we again confirmed the presence of high SEPT5 expression in PCa. Then, we found that increased SEPT5 expression was correlated with higher-grade cancer, higher cancer stage and worse survival in PCa based on the TCGA dataset. We also revealed the staining intensity of SEPT5 in tumour specimens gradually increased with increasing Gleason scores based on tissue array data. Taken together, these results indicate that SEPT5 may be a prognostic biomarker of PCa. Then, we investigated the effect of SEPT5 on PCa progression using a mouse allograft animal model, and the results demonstrated that SEPT5 downregulation significantly inhibited the growth of PCa cells.

After determining the inhibitory effect of SEPT5 on PCa progression, we were curious about the underlying mechanism. It has been reported that SEPT7 can remodel the cytoskeleton by affecting cellular plasticity and deformability, which affects the migratory behaviour of T cells 41, 42. In addition, immune cell development and function can be regulated by the SEPTIN family. We wondered whether SEPT5 exerts its protumor effect by regulating immune cells 43, 44; thus, we first explored the relationship between SEPT5 expression and diverse immune infiltration levels in PCa based on the TCGA dataset. The expression of SEPT5 was found to be significantly negatively correlated with a series of immune cells, such as CD4^+^ T cells, CD8^+^ T cells, and NK cells. In addition, we found that there was a higher abundance of major immune cells in the low SEPT5 expression group than in the high SEPT5 expression group. Finally, we verified the effect of SEPT5 on major immune cells, such as CD4^+^ T cells, CD8^+^ T cells, NK cells, B cells, MDSCs and macrophages. We found that the proportion of NK cells increased after SEPT5 knockdown. The level of NK cell infiltration has been shown to be inversely correlated with the clinical outcome of PCa biopsy 45, and NK cells decrease after tumour progression 46. These studies are consistent with our findings. In addition, CD8^+^ T cells play a crucial role in antitumor immunity reactivated by immunotherapies in patients with PCa 6. Intratumoral CD8^+^ T cell infiltration was significantly reduced in PCa tumour tissues 47 and independently associated with improved survival in PCa patients 48. Therefore, we focused on the effect of SEPT5 on CD8^+^ T cells. In the present study, we confirmed that SEPT5 expression was significantly negatively correlated with CD8^+^ T cell infiltration in PCa patients and SEPT5 knockdown could increase the proportion and activation of CD8^+^ T cells in PCa. Together, our findings suggest that SEPT5 may play a protumor role by inhibiting antitumor immune cells. However, the only limitation is that we were not able to detect which subpopulations of CD8^+^ T cells were changed after SEPT5 knockout in our research.

Next, we investigated the mechanism by which SEPT5 affects immune cells, especially CD8^+^ T cells. The antitumor function of CD8^+^ T cells is highly dependent on two key factors: CD8^+^ T cell infiltration into the tumour site and CD8^+^ T cell differentiation and activation. Chemokines are known for their ability to stimulate cell migration and play a critical role in immune cell infiltration 49. Therefore, we explored the correlation between chemokines and SEPT5 expression, especially the chemokines associated with CD8^+^ T cell recruitment. CCL2, CCL4, CCL5, CCL7, CCL20, CCL23, CXCL5, CXCL6, CXCL9, CXCL10, CXCL13, and CX3CL1 are involved in improving CD8^+^ T cell trafficking and localization in tumour sites 50-52. In this study, we found that a significant negative correlation between SEPT5 expression and chemokine gene expression associated with CD8^+^ T cell recruitment based on bioinformatics analysis. The results from the RNA-Seq analysis showed higher expression of CCL5, CXCL5, CXCL9 and CXCL10 after SEPT5 knockdown, and those results were also comfirmed by real time PCR and chemokine array. These chemokines are positively correlated with CD8^+^ T cell infiltration in the TME 53. These results suggest that SEPT5 may reduce CD8^+^ T cells infiltration by inhibiting the expression of chemokine genes associated with CD8^+^ T cell recruitment, such as CCL5, CXCL5, CXCL9 and CXCL10. INFG is an important regulator for those chemokines, the production of CCL5, CXCL9 and CXCL10 is dependent on IFNG 54, 55. IFNGR1 is the ligand-binding subunit which binds IFNG with high affinity, and CXCL9 and CXCL10 induction is dependent on IFNGR 56. In this study, we found that SEPT5 expression was significantly negatively correlated with IFNGR1 expression, and SEPT5 knockdown could increase the expression of IFNGR1 in mRNA and protein levels. In brief, SEPT5 may inhibit IFNG-inducible chemokines by inactivating IFNG/IFNGR axis in PCa.

We also found that downregulation of SEPT5 promoted the expression of GZMA, which is evidence of CD8^+^ T cell activation 57. CD8^+^ T cell differentiation and activation are associated with cytokines. Therefore, we explored the correlation between cytokines and SEPT5 expression, especially cytokines associated with CD8^+^ T cell activation, such as IL-1, IL-6, and IL-18 52, 58, 59. The expression levels were verified, and the results indicate that SEPT5 may suppress the activation of CD8^+^ T cells by inhibiting the expression of cytokines associated with CD8^+^ T cell activation.

## Conclusion

In summary, SEPT5 expression differs between normal tissue and tumour tissues in multiple cancer types, and SEPT5 is highly expressed in PCa. Higher SEPT expression is positively correlated with poor prognosis in PCa. SEPT5 expression is correlated with infiltration of various types of immune cells, such as B cells, CD4^+^ T cells, CD8^+^ T cells, and macrophages. Downregulation of SEPT5 can inhibit the progression of PCa, possibly by increasing immune cell infiltration levels, especially CD8^+^ T cells, through promotion of IFNG-inducible chemokines (CCL5, CXCL5, CXCL9, CXCL10) and cytokine gene expression (IL-1, IL-6, IL-7, IL-12, etc) associated with immune cells.

## Supplementary Material

Supplementary figures and tables.

## Figures and Tables

**Table 1:** Correlation analysis between SEPT5 expression and immune cell infiltration levels in TISIDB database.

	rho	P value
Activated CD8 cells	-0.031	0.487
**Central memory CD8 T cells**	**-0.365**	**2.41e-17**
**Effector memory CD8 T cells**	**-0.332**	**4.02e-14**
**Activated CD4 T cells**	**-0.345**	**3.13e-15**
**Central memory CD4 T cells**	**-0.314**	**9.35e-13**
**Effector memory CD4 T cells**	**-0.371**	**2.2e-16**
**T follicular helper cells**	**-0.286**	**9.18e-11**
**Gamma delta T cells**	**-0.176**	**7.73e-05**
**Type 1 T helper cells**	**-0.358**	**1.59e-16**
**Type 17 T helper cells**	**-0.384**	**2.2e-16**
**Type 2 T helper cells**	**-0.495**	**2.2e-16**
**Regulatory T helper cells**	**-0.299**	**1.19e-11**
**Activated B cells**	**-0.351**	**8.42e-16**
**Immature B cells**	**-0.41**	**2.2e-16**
**Memory B cells**	**-0.443**	**2.2e-16**
**Natural killer cells**	**-0.399**	**2.2e-16**
CD56bright natural killer cells	0.074	0.0971
**CD56dim natural killer cells**	**0.26**	**4.26e-09**
**Myeloid derived suppressor cells**	**-0.233**	**1.65e-07**
**Natural killer T cells**	**-0.369**	**2.98e-18**
**Activated dendritic cells**	**-0.201**	**6.38e-06**
Plasmacytoid dendritic cells	-0.01	0.819
**Immature dendritic cells**	**-0.396**	**2.2e-16**
**Macrophages**	**-0.229**	**2.68e-07**
**Eosinophils**	**-0.355**	**3.34e-16**
**Mast cells**	**-0.256**	**7.10e-09**
**Monocytes**	**0.265**	**2.1e-09**
**Neutrophils**	**-0.252**	**1.3e-08**

**Table 2:** The proportion of immune cells detected by CyTOF.

Cell type	% of living cells		% of CD45+ cells	
	SEPT5-Vector (n = 4)	SEPT5-KD (n = 4)	SEPT5-Vector (n = 4)	SEPT5-KD (n = 4)
CD45	13.05 ± 1.90	92.82 ± 1.74***		
CD326	13.42 ± 1.84	0.03 ± 0.01***		
CD8^+^ T cells	2.12 ± 0.43	35.82 ± 3.16**	15.89 ± 1.07	38.45 ± 2.77***
B cells	2.36 ± 0.20	1.77 ± 0.20	19.03 ± 2.46	1.91 ± 0.22**
Plasma cells	0.42 ± 0.13	0.50 ± 0.04	2.98 ± 0.57	0.54 ± 0.04*
NK cells	0.05 ± 0.01	1.00 ± 0.25*	0.42 ± 0.09	1.08 ± 0.29
Macrophages	0.43 ± 0.02	2.69 ± 0.33**	3.45 ± 0.38	2.88 ± 0.33
MDSCs	1.39 ± 0.18	2.97 ± 0.25**	11.76 ± 2.86	3.19 ± 0.23
CD4^+^ T cells	0.41 ± 0.12	3.67 ± 0.08***	2.96 ± 0.55	3.96 ± 0.15***

Notes: Compared with the SEPT5-vector group; *, p < 0.05; **, p < 0.01; ***, p < 0.001
