# Supplementary material for: Downregulation of SEPTIN5 inhibits prostate cancer progression by increasing CD8+ T cell infiltration
Source: Int J Biol Sci. 2022 Oct 17;18(16):6035–51. doi: 10.7150/ijbs.76573 (PMC9682533; doi:10.7150/ijbs.76573)
Supplement: Supplementary file 1 — Supplementary figures and tables. [file ijbsv18p6035s1.pdf]

Supplementary material

1. Supplementary Figures

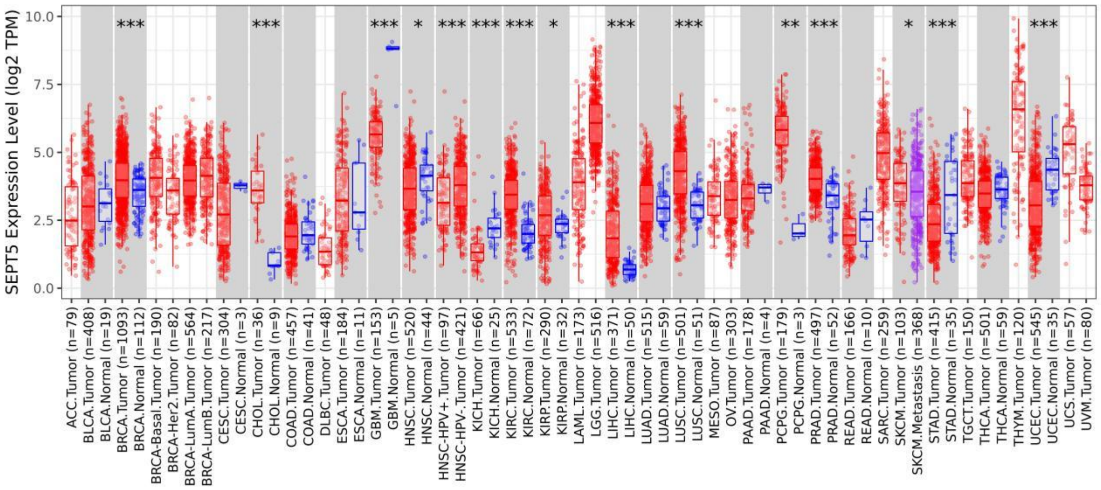

Fig. S1. Aberrant expression of SEPT5 in tumor based on TCGA dataset.

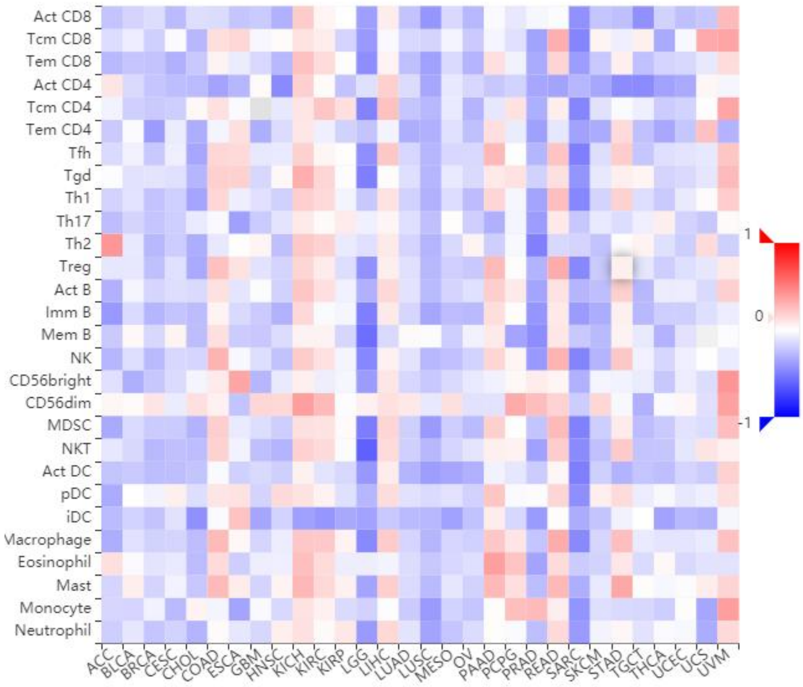

Fig. S2. Correlation between SEPT5 expression and 28 types of immune cell infiltration.

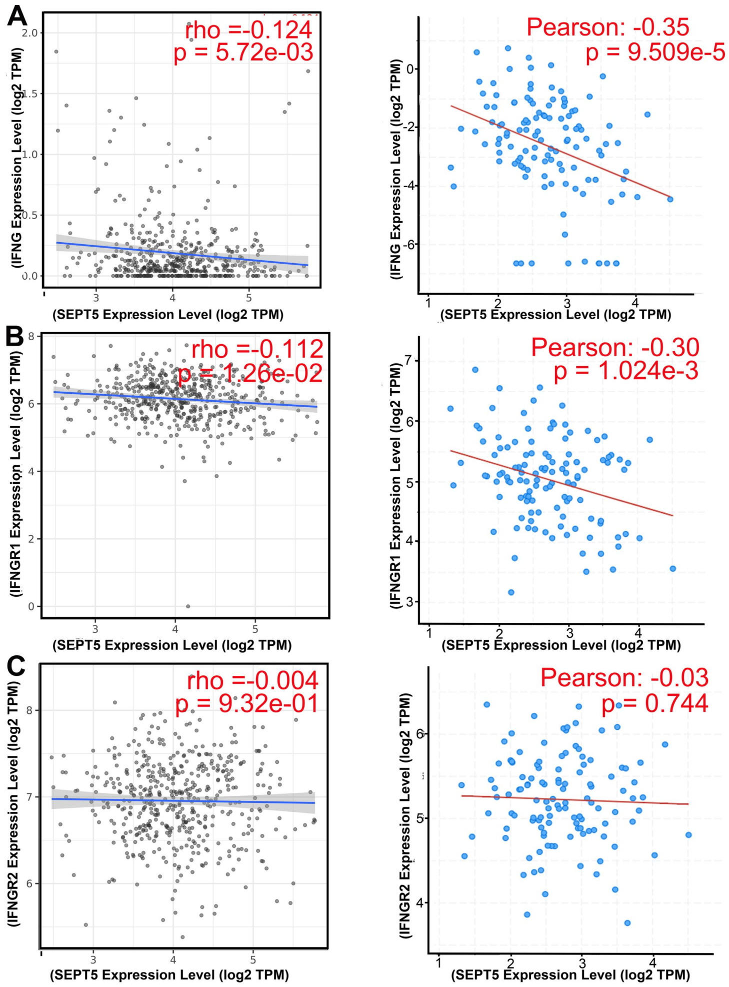

Fig. S3. SEPT5 expression may be significantly negatively correlated with IFN-γ/IFN-γR axis in PCa. The correlation between the IFNG (A), IFNGR1(B), IFNGR2 (C) expression and SEPT5 expression in PCa tissues was analyzed based on TCGA dataset and DKFZ dataset.

2. Supplementary Tables

Table. S1 Antibodies used in this study

Assay	Antibody	Company	Cat#	Dilution	Metal-labeled
WB	SEPT5	Proteintech	11631-1-AP	1:2000	
	HRP-conjugated Tubulin	Proteintech	HRP-66031	1:5000	
	IFNGR1	Proteintech	10808-1-AP	1:1000	
IHC	SEPT5	Proteintech	11631-1-AP	1:200	
	Ki67	Abcam	ab15580	1:400	
	CD8a	Sino Biological	50389-T26	1:200	
	Granzyme A	Proteintech	11288-1-AP	1:100	
	CD8	Proteintech	66868-1-Ig	1:20000	
CyTOF	CD45	Fluidigm Sciences	3089005B	1:100	89Y
	B220	Fluidigm Sciences	3144011B	1:100	144Nd
	BCMA	R&D Systems	AF593	1:200	166Er
	CD3	Fluidigm Sciences	3152004B	1:100	152Sm
	CD4	Fluidigm Sciences	3145002B	1:100	145Nd
	CD8a	Fluidigm Sciences	3168003B	1:100	168Er
	CD335	Fluidigm Sciences	3153006B	1:100	153Eu
	CD326	Fluidigm Sciences	3165014B	1:100	165Ho
	CD11b	Fluidigm Sciences	3172012B	1:100	172Yb
	Gr1	Fluidigm Sciences	3141005B	1:100	141Pr
	F4/80	Fluidigm Sciences	3146008B	1:100	146Nd

Table S2. qPCR primers used in this study.

Species	Name		Sequence(5' - 3')
Mus musculus	**SEPT5**	Forward	GAAAGGTTTCGACTTCACGCT
		Reverse	CCGGTCCTTATACAGGTCGGT
	**Actin**	Forward	CGTTGACATCCGTAAAGACC
		Reverse	AACAGTCCGCCTAGAAGCAC
	**CCL5**	Forward	GCTGCTTTGCCTACCTCTCC
		Reverse	TCGAGTGACAAACACGACTGC
	**CXCL5**	Forward	TCCAGCTCGCCATTCATGC
		Reverse	TTGCGGCTATGACTGAGGAAG
	**CXCL9**	Forward	TCCTTTTGGGCATCATCTTCC
		Reverse	TTTGTAGTGGATCGTGCCTCG
	**CXCL10**	Forward	CCAAGTGCTGCCGTCATTTTC
		Reverse	GGCTCGCAGGGATGATTTCAA
	**IFNGR1**	Forward	CTTGAACCCTGTCGTATGCTGG
		Reverse	TTGGTGCAGGAATCAGTCCAGG
Homo sapiens	**SEPT5**	Forward	CGCATCAGCCAGACGGTAG
		Reverse	CCGCTCTCATCACGGAAGT
	**Actin**	Forward	CATGTACGTTGCTATCCAGGC
		Reverse	CTCCTTAATGTCACGCACGAT
	**CCL5**	Forward	TGTACTCCCGAACCCATTTC
		Reverse	TACACCAGTGGCAAGTGCTC
	**CXCL5**	Forward	AGCTGCGTTGCGTTTGTTTAC
		Reverse	TGGCGAACACTTGCAGATTAC
	**CXCL9**	Forward	CCAGTAGTGAGAAAGGGTCGC
		Reverse	AGGGCTTGGGGCAAATTGTT
	**CXCL10**	Forward	GTGGCATTCAAGGAGTACCTC
		Reverse	TGATGGCCTTCGATTCTGGATT
	**IFNGR1**	Forward	GAGACGAGCAGGAAGTCGAT
		Reverse	CATCTTCCTTCTGCGTGAGT

Table S3. Cell_abundance between SEPT5 low group and SEPT5 high group based on TCGA dataset

Cell type	Cell_abundance	
	SET5_low	SET5_high
**Act_CD8**	-0.004 ± 0.02082	-0.0241 ± 0.02246
**Tcm_CD8**	**0.076 ± 0.01521**	**-0.0671 ± 0.01583**
**Tem_CD8**	**0.115 ± 0.02451**	**-0.0835 ± 0.02514**
**Act_CD4**	**0.0833 ± 0.02362**	**-0.1086 ± 0.02216**
**Tcm_CD4**	**0.0706 ± 0.01506**	**-0.061 ± 0.01537**
**Tem_CD4**	**0.065 ± 0.01352**	**-0.0604 ± 0.01322**
**Tfh**	**0.065 ± 0.01775**	**-0.061 ± 0.0179**
**Tgd**	**0.0277 ± 0.01216**	**-0.0293 ± 0.01332**
**Th1**	**0.0983 ± 0.01833**	**-0.714 ± 0.01814**
**Th17**	**0.099 ± 0.01494**	**-0.0603 ± 0.0158**
**Th2**	**0.1268 ± 0.01607**	**-0.0918 ± 0.01696**
**Treg**	**0.0829 ± 0.02545**	**-0.0835 ± 0.02419**
**Act_B**	**0.1214 ± 0.02723**	**-0.1331 ± 0.02605**
**Imm_B**	**0.129 ± 0.02488**	**-0.1397 ± 0.02494**
**Mem_B**	**0.1312 ± 0.02043**	**-0.0978 ± 0.01952**
**NK**	**0.1156 ± 0.01675**	**-0.0785 ± 0.01675**
CD56bright	-0.0046 ± 0.0117	0.0085 ± 0.01162
**CD56dim**	**-0.0662 ± 0.0164**	**0.0629 ± 0.01563**
**MDSC**	**0.0791 ± 0.02815**	**-0.082 ± 0.02901**
**NKT**	**0.097 ± 0.01828**	**-0.808 ± 0.01907**
**Act_DC**	**0.097 ± 0.01828**	**-0.0808 ± 0.01907**
pDC	0.0124 ± 0.01055	0.005 ± 0.01047
**iDC**	**0.0876 ± 0.01358**	**-0.0641 ± 0.01327**
**Macrophage**	**0.0606 ± 0.01936**	**-0.0451 ± 0.01974**
**Eosinophil**	**0.1015 ± 0.0218**	**-0.0755 ± 0.01979**
**Mast**	**0.0846 ± 0.02175**	**-0.0497 ± 0.02274**
**Monocyte**	**-0.0622 ± 0.01598**	**0.0384 ± 0.01469**
**Neutrophil**	**0.0702 ± 0.02055**	**-0.0536 ± 0.01872**

Table S4. Correlation analysis between SEPT5 expression and chemokine-related gene expression based on TCGA dataset

infiltrates	rho	p value
CCL1	0.015977186	0.722086048
**CCL2**	**-0.204376803**	**4.27E-06**
CCL3	-0.068212787	0.128466639
**CCL4**	**-0.143145713**	**0.001360468**
**CCL5**	**-0.234116824**	**1.26E-07**
**CCL7**	**-0.129669961**	**0.003747382**
CCL8	-0.084497647	0.059527838
CCL11	-0.082055452	0.067305707
**CCL13**	**-0.088463349**	**0.048489957**
**CCL14**	**-0.145979336**	**0.001086963**
CCL15	-0.064404097	0.15125439
CCL16	-0.012228713	0.785453398
**CCL17**	**-0.139425697**	**0.001815648**
CCL18	-0.072704525	0.105117208
**CCL19**	**-0.166191004**	**0.000195066**
**CCL20**	**-0.247769253**	**2.11E-08**
**CCL21**	**-0.136146866**	**0.002328384**
**CCL22**	**-0.213979971**	**1.44E-06**
**CCL23**	**-0.129244522**	**0.003863609**
CCL24	-0.028597049	0.524323003
CCL24	-0.028597049	0.524323003
**CCL25**	**0.089406234**	**0.046134235**
CCL26	0.0485633	0.279408381
CCL27	0.055548734	0.215915883
**CCL28**	**-0.237089442**	**8.60E-08**
**CXCL1**	**-0.237999767**	**7.65E-08**
**CXCL2**	**-0.235817348**	**1.01E-07**
**CXCL3**	**-0.24882309**	**1.83E-08**
**CXCL5**	**-0.367121668**	**2.46E-17**
**CXCL6**	**-0.346495371**	**1.71E-15**
**CXCL9**	**-0.098625075**	**0.027754034**
**CXCL10**	**-0.104057865**	**0.020199614**
CXCL11	-0.060134572	0.180310909
**CXCL12**	**-0.205399332**	**3.81E-06**
**CXCL13**	**-0.202369621**	**5.32E-06**
CXCL14	-0.047713813	0.287914873
**CXCL16**	**-0.128477216**	**0.004081516**
**CXCL17**	**-0.292130185**	**2.96E-11**
**CX3CL1**	**-0.26506952**	**1.88E-09**

Table S5. Correlation analysis between SEPT5 expression and cytokine-related gene expression based on TCGA dataset

infiltrates	rho	p value
IL1A	-0.051261379	0.253528582
**IL1B**	**-0.18990156**	**1.99E-05**
**IL2**	**-0.092237493**	**0.039630529**
IL3	0.060205925	0.179793535
IL4	0.044112995	0.325889888
IL5	-0.013208842	0.76872775
**IL6**	**-0.178510246**	**6.18E-05**
**IL7**	**-0.319684483**	**2.70E-13**
**IL8**	**-0.257158617**	**5.80E-09**
IL9	-0.031261827	0.486395477
**IL10**	**-0.164458794**	**0.000227849**
**IL11**	**0.112313257**	**0.012141172**
**IL12A**	**-0.145143708**	**0.001161822**
**IL12B**	**-0.119733346**	**0.007475749**
IL13	-0.019306572	0.667340283
**IL15**	**-0.212543824**	**1.70E-06**
**IL16**	**-0.21725399**	**9.85E-07**
**IL17A**	**-0.212738572**	**1.66E-06**
IL17B	-0.069461903	0.121602845
IL17C	0.026266678	0.558687027
**IL17D**	**0.169343758**	**0.000146445**
**IL17F**	**-0.151920628**	**0.000670192**
**IL18**	**-0.227929763**	**2.73E-07**
IL19	-0.079838595	0.075070021
**IL20**	**-0.132989237**	**0.002943891**
IL21	-0.051310818	0.253070428
**IL22**	**-0.124249756**	**0.005493993**
**IL23A**	**-0.179367583**	**5.69E-05**
IL28A	-0.001150015	0.97957706
IL28B	0.049140268	0.273728915
**IL24**	**-0.166592149**	**0.000188132**
**IL25**	**-0.130273179**	**0.003588015**
IL26	-0.085004581	0.058010297
IL27	-0.004960855	0.91206956
IL29	-0.04000638	0.372987047
**IL31**	**0.097932484**	**0.028873012**
**IL32**	**-0.10747164**	**0.016428534**
**IL33**	**-0.327294379**	**6.74E-14**

IL34	-0.08412544	0.060662846
**TGFA**	**-0.263539029**	**2.34E-09**
TGFB1	-0.022659851	0.613931618
**TGFB2**	**-0.31905439**	**3.02E-13**
**TGFB3**	**-0.238476954**	**7.19E-08**
**CSF1**	**-0.231401835**	**1.77E-07**
CSF2	-0.06002218	0.181128079
**CSF3**	**-0.180194381**	**5.25E-05**
**TNF**	**-0.186658672**	**2.77E-05**
**IFNG**	**-0.123674714**	**0.005716833**

Table S6. The expression of cytokine gene beween SEPT5-vector group and SEPT5-KD group according to RNA-seq results

Symbol	FPKM		p value
	Vector	SEPT5 knockdown	
**IL1A**	**0.76 ± 0.18**	**15.74 ± 2.68**	**0. 03**
**IL1B**	**292.39 ± 17.18**	**1802.79 ± 43.02**	**0**
IL1F10	0.01 ± 0.01	0.1 ± 0.05	0. 179
**IL6**	**37.54 ± 2.77**	**153.82 ± 10.99**	**0. 001**
**IL7**	**0.17 ± 0.04**	**0.76 ± 0.18**	**0. 032**
IL11	6.75 ± 0.71	7.82 ± 0.19	0. 218
**IL12A**	**0.45 ± 0.12**	**1.61 ± 0.1**	**0. 002**
**IL15**	**3.68 ± 0.32**	**5.17 ± 0.37**	**0. 039**
IL16	0.01 ± 0.01	0.02 ± 0.01	0. 326
IL17C	0.12 ± 0.06	0.06 ± 0.05	0. 483
IL17D	11.18 ± 0.87	11.23 ± 0.36	0. 958
**IL18**	**40.61 ± 1.35**	**58.99 ± 2**	**0. 002**
**IL19**	**0 ± 0**	**0.25 ± 0.08**	**0**
IL20	0.01 ± 0.01	0.4 ± 0.13	0. 089
**IL23A**	**24.82 ± 0.77**	**24.51 ± 1**	**0. 039**
**IL24**	**0.66 ± 0.16**	**13.16 ± 0.43**	**0. 001**
**IL27**	**0 ± 0**	**0.04 ± 0.02**	**0**
IL32	75.83 ± 5.21	124.47 ± 3.87	0. 158
**IL33**	**0.01 ± 0.01**	**0.04 ± 0.01**	**0. 002**
IL34	0.05 ± 0.05	0.04 ± 0.02	0. 116
IL36A	0.02 ± 0.02	0 ± 0	0. 904
IL36B	0.11 ± 0.09	0 ± 0	0. 374
**IL36G**	**0.19 ± 0.04**	**0.57 ± 0.04**	**0. 002**
**IL37**	**11.85 ± 0.15**	**113.2 ± 4.95**	**0**
**TNF**	**0.65 ± 0.06**	**4.23 ± 0.12**	**0**
**CSF1**	**2.68 ± 0.13**	**5.09 ± 0.58**	**0. 016**
**CSF2**	**29.11 ± 0.72**	**136.55 ± 4.3**	**0**
CSF3	13.5 ± 1.46	14.32 ± 1.07	0. 674
VEGFA	38.12 ± 2.15	43.34 ± 2.64	0. 2
**VEGFB**	**28.69 ± 1.12**	**41.53 ± 1.44**	**0. 002**
**VEGFC**	**15.65 ± 0.73**	**22.84 ± 0.35**	**0. 001**
**TGFA**	**12.38 ± 1.37**	**19.68 ± 0.45**	**0. 007**
TGFB1	32.45 ± 0.58	37.97 ± 1.33	0. 059
**TGFB1I1**	**4.96 ± 0.61**	**8.27 ± 0.74**	**0. 026**
**TGFB2**	**8.16 ± 0.52**	**15.45 ± 1.22**	**0. 005**
TGFB3	1.62 ± 0.15	1.4 ± 0.06	0. 249

Table S7. The expression of chemokine gene beween SEPT5-vector group and SEPT5-KD group according to RNA-seq results

Symbol	FPKM		
	Vector	SEPT5 knockdown	p value
CCL2	0.02 ± 0.02	0.13 ± 0.13	0.488
CCL3	0 ± 0	0.13 ± 0.05	0.058
**CCL3L1**	**0 ± 0**	**0.2 ± 0.06**	**0.031**
CCL4	0 ± 0	0.06 ± 0.06	0.374
**CCL5**	**0.47 ± 0.1**	**54.97 ± 2.19**	**0.002**
CCL17	0 ± 0	0.08 ± 0.04	0.152
**CCL22**	**0.03 ± 0.02**	**1.91 ± 0.14**	**0.005**
CCL24	0.51 ± 0.21	0.45 ± 0.06	0.081
**CCL25**	**0.02 ± 0.02**	**0.12 ± 0.02**	**0.025**
CCL26	4.67 ± 0.67	4.17 ± 0.46	0.574
CCL28	4.38 ± 0.35	4.97 ± 0.44	0.347
CX3CL1	0 ± 0	0.03 ± 0.01	0.114
CXCL1	302.82 ± 19.49	313.95 ± 24.62	0.741
**CXCL2**	**12.81 ± 0.81**	**15.22 ± 0.67**	**0.082**
**CXCL3**	**110.21 ± 5.14**	**54.42 ± 3.39**	**0.001**
**CXCL5**	**121.17 ± 5.56**	**199.96 ± 11.03**	**0.003**
CXCL6	46.94 ± 2.79	50.75 ± 0.95	0.266
**CXCL8**	**443.41 ± 26.78**	**723.56 ± 31.02**	**0.002**
**CXCL9**	**0 ± 0**	**0.19 ± 0.03**	**0.002**
**CXCL10**	**0.04 ± 0.02**	**5.51 ± 0.5**	**0.000**
**CXCL11**	**0.05 ± 0.03**	**3.79 ± 0.74**	**0.037**
CXCL12	0 ± 0	0.03 ± 0.03	0.423
**CXCL16**	**39.13 ± 0.66**	**46.98 ± 1.53**	**0.009**
CXCL17	0.11 ± 0.07	0.01 ± 0.01	0.263
XCL1	0 ± 0	0.010.01	0.374
